# Basal and longitudinal changes in serum levels of TSH in morbid obese patients experiencing failure or success of dietary treatment

**DOI:** 10.1007/s40519-020-01043-x

**Published:** 2020-10-17

**Authors:** Laura Croce, Cristina Pallavicini, Silvia Crotti, Francesca Coperchini, Linda Minnelli, Flavia Magri, Luca Chiovato, Mario Rotondi

**Affiliations:** 1 Unit of Internal Medicine and Endocrinology, Laboratory for Endocrine Disruptors, Department of Internal Medicine and Therapeutics, Istituti Clinici Scientifici Maugeri IRCCS, University of Pavia, Via S. Maugeri 4, 27100 Pavia, Italy; 2 Department of Internal Medicine and Therapeutics, University of Pavia, 27100 Pavia, Italy; 3 PhD Course in Experimental Medicine, University of Pavia, 27100 Pavia, Italy

**Keywords:** Obesity, Thyroid, Weight loss, Diet

## Abstract

**Purpose:**

The relationship between thyroid function and obesity is a widely investigated one. The impact of thyroid hormones in determining the outcome of dietary/lifestyle interventions remains to be fully elucidated. The aim of this study was to compare basal and post dietary-intervention circulating thyroid-function parameters, lipid profile and fasting-glucose in euthyroid obese patients according to a success or failure of a dietary intervention program.

**Methods:**

In a retrospective longitudinal case–control study we enrolled 50 euthyroid obese patients who experienced a success in dietary intervention, as defined by a BMI reduction of at least 5% from baseline (Success Group) and 50 sex and age-matched euthyroid obese patients who experienced failure in dietary intervention as defined by either stable or increased body weight throughout the follow-up (Failure Group). Serum thyroid function parameters and metabolic profile at baseline and at the end of follow-up were collected.

**Results:**

At baseline, the two groups showed similar BMI, total-cholesterol, HDL-cholesterol and fasting-blood-glucose, but patients in Success Group had a significantly higher TSH as compared with Failure Group (2.20 ± 0.97 vs 1.66 ± 0.73, respectively, *p* < 0.001). Throughout a mean follow-up of 21.4 months TSH significantly decreased in Success Group (2.20 ± 0.97 vs 2.06 ± 0.98; *p* = 0.029) and increased in Failure Group (1.63 ± 0.72 vs 2.01 ± 0.99; *p* < 0.001). Multiple regression analysis showed that the outcome of the dietary intervention was significantly and independently related to baseline BMI (0.925; 0.861–0.993), age (0.957; 0.922–0.993), TSH (0.531; 0.290–0.973) and TSH-changes (1.011; 1.000–1.022) during follow-up.

**Conclusions:**

Baseline serum TSH level is related to the final outcome of a dietary intervention program in obese patients.

**Level of evidence III:**

Evidence obtained from a retrospective cohort or case–control analytic studies.

## Introduction

A moderate elevation of TSH concentrations, often associated with triiodothyronine (T3) values in or slightly above the upper normal range, is frequently found in obese humans [1, 2]. This observation together with the notion that T3 is involved in the regulation of basal energy expenditure, lead to the hypothesis that small differences in thyroid function, even in the presence of euthyroidism, could influence body weight status in the long term [2, 3]. Thus, thyroid status has been regarded as one of the factors involved in the pathogenesis of the obesity epidemics. Although this hypothesis is a long-standing one, in the last years a huge body of evidence supported an opposite theory, namely that these slight thyroid alterations could be a consequence rather than a cause of obesity [4]. Indeed, in a specifically designed study from our group, it was shown that morbid obese patients display a high rate of isolated hyperthyrotropinemia not associated with the typical features of autoimmune hypothyroidism. In particular, the hyperthyrotropinemia of obese patients was characterized by: (1) low prevalence of positive tests for thyroid antibodies; (2) lack of female gender prevalence; and (3) similar free T4 (FT4)/free T3 (FT3) ratio as compared to obese patients with normal serum levels of TSH [5]. In addition, in a study comparing the circulating lipid profile between morbid obese patients and non-obese patients with raised serum levels of TSH, despite similar serum levels of TSH, FT4 and FT3, morbid-obese patients displayed significantly lower mean levels of total cholesterol, further supporting the concept that these latter might not be truly hypothyroid [6].

It seems worth highlighting that, according to the NHANES III survey, the prevalence of positive tests for thyroid antibodies was similar between patients with morbid obesity and normal serum TSH as compared with the general population [7].

Hyperthyreotropinemia related to obesity has been shown to be reversible after weight loss, either obtained with diet [8–11] or bariatric surgery [12, 13].

In particular, Reinehr et al. [9] reported that in obese children with similar levels of TSH, undergoing an intensive program of dietary and lifestyle intervention, a significant decrease in TSH levels was observed in those children experiencing weight loss but not in those with stable body weight.

The aim of our study was to compare basal and post dietary intervention circulating thyroid function parameters, lipid profile and fasting glucose in obese patients according to a success or failure of a dietary intervention program.

## Patients and methods

In this study, data were retrospectively collected from morbid obese patients recruited in the Unit of Internal Medicine and Endocrinology at ICS Maugeri Hospital (Pavia, Italy) who had undertaken a Dietary and Behavioural intervention program. Patients were enrolled only if they had experienced a successful intervention program as defined by a BMI reduction of at least 5% from baseline to the last evaluation. A total number of 50 patients (22 males and 28 females) were selected. The control group encompassed an equal number of sex and age-matched patients (22 males and 28 females) in whom failure of the dietary-behavioral intervention program (as defined by either stable or increased body weight throughout the follow-up) was observed.

Common inclusion criteria were: (1) BMI ≥ 35 kg/m^2^; (2) Regular dietistic follow-up for at least 6 months; 3) availability of TSH measurement at the beginning and at the end of the dietistic follow-up; 4) age ≥ 18 years old.

Exclusion criteria for both groups were: (1) current or previous history of thyroid disease or any alteration of thyroid function parameters; (2) current or past use of thyroid function-modifying drugs; (3) previous bariatric surgery.

The length of the follow-up time was calculated for each patient starting from the first measurement of TSH which was performed at diagnosis (T0) to the last TSH measurement (T1).

For all patients, the TSH value measured at the beginning and at the end of the dietistic approach was collected. Serum FT4, total cholesterol, HDL cholesterol, fasting glucose values at the beginning and at the end of the dietary intervention program were collected.

All patients signed an informed consent concerning the future use of their clinic data for research purposes and data collected remained strictly confidential and anonymous, according to the ethical rules of our Hospital institutions and to the Declaration of Helsinki. Formal approval by the ethical committee was not required in accordance with the Italian regulation for non-interventional (observational) retrospective studies concerning human beings (AIFA Guidelines for Observational Studies, see www.AIFA.gov).

### Dietary and behavioral intervention

Eating habits were investigated, through an accurate dietary history collection, using a specially prepared form, referred to 3 typical days (2 weekdays and 1 holiday), the remote food history, or dietary history, provided to understand the setting of the diet at home for individual patients. Based on the results of this enquiry the average calorie intake and the bromatological composition of the patient's usual diet was calculated. Patients also filled in a questionnaire on food consumption frequencies to identify the most frequent errors and provide solutions to better balance their diet. A leaflet on “Healthy and correct nutrition” was delivered, containing the basics of proper nutrition and regular physical activity was given to each patients. This booklet was designed to give an educational aid for continuity at home.

The energy needs were estimated by calculating the basal metabolic rate with the Harris-Benedict formula, multiplied by the appropriate level of physical activity.

Using the nutritional analysis program “Reasoned Diet 3” (supplied by the Clinic of Dietetics and Clinical Nutrition of the Maugeri Scientific Clinical Institutes), a personalized diet was subsequently calculated for each patient. All diets were low caloric diets with an average energy deficit of 700- 800 kcal compared to the estimated expenditure. The dietary scheme was designed respecting the requirements dictated by the LARN (Reference intake levels of nutrients and energy), including 45–55% of carbohidrates (with a content of simple sugars < 15%, that latter could be further restricted, in relation to the patient’s clinical condition, i..e diabetes mellitus, insulin resistance, hypertriglyceridemia, etc.) and 25–35% of lipids (with saturated fats < 10%, monounsaturated 10–15% and polyunsaturated 8–10%). The daily protein intake was 0.8–1 g/kg of desirable weight (desirable weight means a weight corresponding to a BMI of 22.5 kg/m^2^).

The diet was structured around 3 main meals, possibly adding 1 or 2 snacks throughout the day, depending on the patient's needs, respecting the Mediterranean diet model.

### Statistical analysis

Statistical analysis was performed using SPSS software version 20 (SPSS, Inc., Evanston, IL, USA). Between-group comparisons were performed by Student's *t* test for unpaired data and by Mann–Whitney *U *test according to a normal or a nonparametric distribution of the variable tested. Paired samples comparisons were performed by Paired Samples *t *test and by Wilcoxon Rank test according to a normal or a nonparametric distribution of the variable tested. Correlation between two variables was ascertained by Pearson and Spearman's correlation tests, as appropriate. Frequencies among groups were compared by χ^2^ test with Fisher’s correction, when appropriate. A multivariate logistic analysis was constructed with the outcome of dietary intervention as the dependent variable and basal BMI, age, sex, basal TSH, percentage of variation of TSH as covariates. A *p* value < 0.05 was considered statistically significant.

## Results

**Fig. 1:**
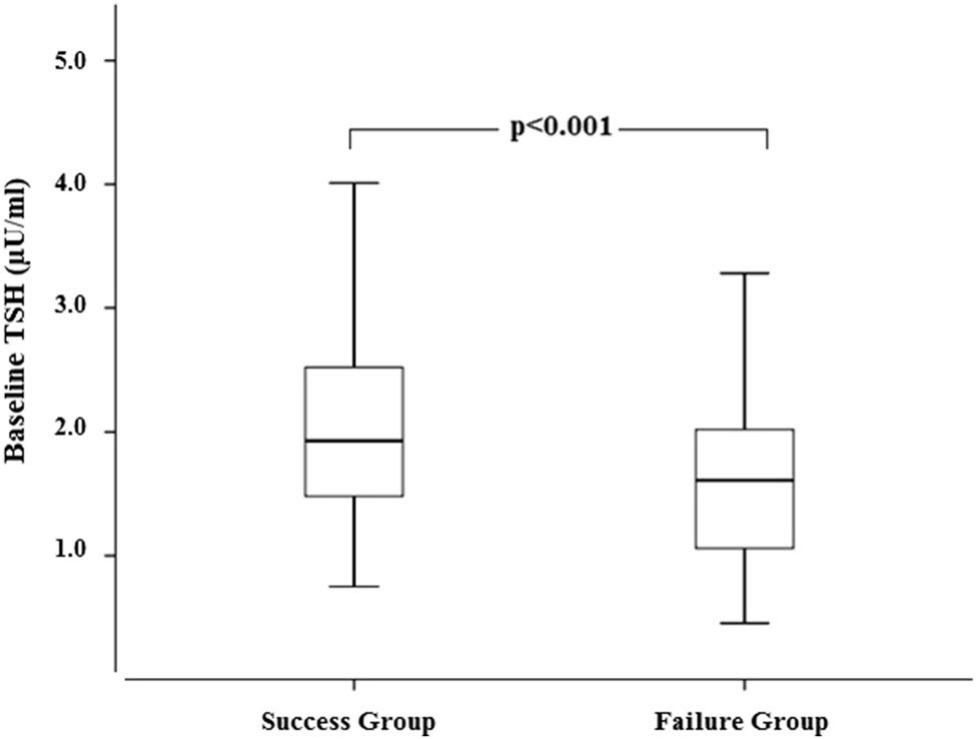
Distribution of basal TSH values in the two groups of patients. TSH basal values were significantly higher in the Success Group compared with the Failure Group [1.93 (0.75–4.57) µU/ml in Success group vs 1.61 (0.46–3.28) µU/ml in Failure group, *p* < 0.001]. Data are expressed as median and 25th and 75th percentiles in boxes and 5th and 95th percentiles as whiskers

The study group encompassed 50 patients in the Success Group and 50 patients in the Failure Group. The anthropometric and biochemical characteristics of the two groups are described in Table 1. The two groups were similar in terms of age (50.01 ± 13.8 in Success Group vs 45.6 ± 13.6 in Failure Group, *p* = 0.111) and baseline BMI (47.6 ± 7.8 in Success Group vs 45.1 ± 5.8 in Failure Group, *p* = 0.080). At T0, patients from Success Group had a significantly higher TSH as compared with Failure Group patients (TSH 2.20 ± 0.9 for Success Group vs 1.66 ± 0.73 for Failure Group, *p* < 0.001) (Fig. 1). Other biochemical parameters including total cholesterol, HDL-cholesterol, and fasting blood glucose levels were similar between the two groups at baseline. 38 patients (17 in the Success Group and 21 in the Failure group, *p* = 0.412) were diabetic. Among these 38 patients, 10 patients were in therapy with insulin (2 in the Success group, 8 in the Failure group, *p* = 0.136), 30 were in therapy with metformin (14 in the Success group, 16 in the failure group, *p* = 0.709), 11 patients were in therapy with a sulfonylurea (2 in the Success Group, 9 in the Failure Group, *p* = 0.070), 5 patients were in therapy with pioglitazone (0 in the Success Group, 5 in the Failure Group, *p* = 0.053), 9 patients were in therapy with a GLP-1 analogue (3 in the success Group, 6 in the Failure Group, *p* = 0.476), 2 patients were in therapy with DPP-IV inhibitors (0 in the success group, 2 in the Failure Group, *p* = 0.492) and 5 patients were in therapy with acarbose (1 in the success group, 4 in the Failure group, *p* = 0.335).

**Table 1:** baseline biochemical and anthropometrical characteristics of the two study groups

	Success group	Failure group	*p* value
Age (years)	50.0 ± 13.8	45.6 ± 13.6	0.111
	50.0 (19.0–80.0)	47.0 (18.0–75.0)	
Sex (M/F)	22/28	22/28	1.000
BMI at baseline (kg/m^2^)	47.7 ± 7.8	45.1 ± 5.8	0.080
	47.2 (35.6–67.9)	43.3 (39.1–62.9)	
TSH (µU/ml)	2.20 ± 0.97	1.66 ± 0.73	< 0.001*
	1.93 (0.75–4.57)	1.61 (0.46–3.28)	
FT4 (ng/dl)	1.08 ± 0.15	1.07 ± 0.17	0.639
	1.03 (0.69–1.45)	1.11 (0.72–1.48)	
Total cholesterol (mg/dl)	185.3 ± 34.0	187.2 ± 32.2	0.773
	185.0 (109.0–271.0)	181.0 (119.0–277.0)	
HDL (mg/dl)	45.6 ± 14.0	48.8 ± 13.7	0.243
	45.0 (21.0–86.0)	48.0 (27.0–91.0)	
Fasting blood glucose (mg/dl)	114.3 ± 38.5	123.9 ± 55.4	0.317
	98.5 (72.0–267.0)	103.0 (77.0–325.0)	

Bold indicates* p* value under 0.05

All data are reported as mean ± standard deviation as well as median(minimum–maximum). Independent samples *T* test was used unless stated otherwise

*Mann–Whitney test

The mean BMI variation and the parallel changes in total cholesterol, HDL-cholesterol, fasting blood glucose and TSH observed from baseline (T0) to the end of follow-up (T1) in the 2 groups are shown in Table 2. As expected, patients in Success Group experienced a significant decrease in BMI (47.6 ± 7.8 at T0 vs 40.7 ± 7.7 kg/m^2^ at T1, *p* < 0.0001) whereas BMI was significantly increased in Failure Group (45.1 ± 5.8 at T0 vs 47.5 ± 5.9 kg/m^2^ at T1, *p* < 0.0001) throughout the study span.

**Table 2:** variation of BMI and biochemical parameters throughout the follow-up in the two study groups

	Success group			Failure group		
	T0	T1	*p* value	T0	T1	*p* value
BMI (kg/m^2^)	47.6 ± 7.8	40.7 ± 7.7	** < 0.0001**	45.1 ± 5.8	47.5 ± 5.9	** < 0.0001**
	47.2 (35.6–67.9)	39.8 (27.7–57.3)		43.3 (39.1–62.9)	46.7 (39.5–67.2)	
TSH (µU/ml)	2.20 ± 0.97	2.06 ± 0.98	0.029*	1.63 ± 0.72	2.01 ± 0.99	0.001*
	1.93 (0.75–4.57)	1.95 (0.24–4.38)		1.61 (0.46–3.28)	1.71 (0.74–5.52)	
FT4 (ng/dl)	1.08 ± 0.15	1.11 ± 0.18	0.220	1.07 ± 0.17	1.09 ± 0.24	0.528
	1.03 (0.69–1.45)	1.11 (0.84–1.46)		1.11 (0.72–1.48)	1.08 (0.67–1.86)	
Total cholesterol (mg/dl)	185.4 ± 34.0	174.6 ± 31.8	**0.007**	187.3 ± 32.2	176.9 ± 32.3	**0.011**
	185.0 (109.0–271.0)	180.0 (79.0–245.0)		181.0 (119.0–277.0)	175.0 (116.0–273.0)	
HDL (mg/dl)	45.6 ± 14.0	45.6 ± 11.4	0.967	48.87 ± 13.7	51.93 ± 14.8	0.051
	45.0 (21.0–86.0)	46.5 (20.0–72.0)		48.0 (27.0–91.0)	52 (25.0–91.0)	
Fasting blood glucose (mg/dl)	114.3 ± 38.5	106.9 ± 25.1	0.059	123.9 ± 55.4	131.9 ± 68.1	0.388
	98.5 (72.0–267.0)	97.5 (79.0–187.0)		103.0 (77.0–325.0)	106.0 (65.0–367.0)	

Bold indicates* p* values under 0.05

All data are reported as mean ± standard deviation as well as median(minimum–maximum). Paired samples *T *test was used unless stated otherwise

*Wilcoxon rank test

While a significant decrease in total cholesterol occurred in both groups, no changes in the circulating levels of HDL-cholesterol nor fasting blood glucose were observed from T0 to T1 in any of the groups. Noteworthy, the longitudinal behavior of the serum levels of TSH was strikingly different between the 2 groups. Indeed, while a slight although a significant decrease in serum TSH was observed from T0 to T1 (2.20 ± 0.9 vs 2.06 ± 0.9, respectively; *p* = 0.029) in Success Group, an opposite and still significant trend was observed in Failure Group (1.63 ± 0.7 in T0 vs 2.01 ± 0.9 in T1, *p* < 0.001). No significant modifications in serum FT4 levels were observed in both groups (1.08 ± 0.15 at T0 vs 1.11 ± 0.18 at T1 in Success Group, *p* = 0.220; 1.07 ± 0.17 at T0 vs 1.09 ± 0.24 at T1 in Failure Group, *p* = 0.528).

The mean follow-up time was similar between the two groups (20.5 ± 22.0 months for Success Group vs 22.3 ± 19.9 months for Failure Group; *p* = 0.656).

To evaluate factors potentially associated with the different outcome of dietary intervention program, a logistic regression model was constructed entering intervention outcome (success/failure) as a dependent variable and baseline BMI, sex, age, baseline TSH, TSH percentage of variation throughout the follow-up as covariates. As shown in Table 3, baseline BMI, age, levels of TSH at T0 and TSH changes (expressed as percentages of the TSH at T0) were independently and significantly related to the outcome of the dietary intervention.

**Table 3:** Logistic regression analysis entering intervention outcome (success/failure) as dependent variable and baseline BMI, sex, age, baseline TSH

	*p* value	Exp (B)	95% CI for EXP (B)	
			Lower	Upper
Age (years)	**0.020**	0.957	0.922	0.993
Basal BMI (kg/m^2^)	**0.031**	0.925	0.861	0.993
Baseline TSH (µU/ml)	**0.041**	0.531	0.290	0.973
Percentage of variation of TSH (%)	**0.045**	1.011	1.000	1.022
Sex (M/F)	0.840	1.099	0.440	2.743

Bold indicates* p* values under 0.05

TSH percentage of variation throughout the follow-up as covariates

## Discussion

The present study was specifically designed to compare changes in circulating thyroid function parameters in euthyroid obese patients experiencing a different outcome of a dietary intervention program. Obese patients were specifically recruited according to either a successful outcome (as defined by a BMI reduction of at least 5%) or failure of the interventional program (as defined by stable and/or increased BMI).

The two groups did not differ in any anthropometric and biochemical parameter at baseline, with the exception of a higher level of TSH which characterized patients of Success Group. The longitudinal evaluation highlighted an opposite trend in TSH behavior according to a different outcome. Indeed, in patients experiencing weight loss, a significant reduction of the circulating TSH concentrations occurred while in patients experiencing failure of the dietary program, a significant increase in serum TSH levels was observed. The multivariate regression model confirmed that baseline levels of TSH, TSH changes during the follow-up, age and basal BMI were all independently and significantly related to the outcome of the dietary intervention.

The findings that both basal TSH levels and its changes throughout the follow-up were found to be related to the different outcome of the dietary intervention program might require to be discussed separately.

The here reported direct relationship between changes in TSH and in BMI in obese patients is in line with previous studies showing that obese patients display higher TSH levels as compared to normo-weight subjects. Reinehr et al. [9], previously reported a significant reduction of TSH levels only in children who obtained a significant weight loss after a 1 yr treatment with hypocaloric diet. At difference with our findings, no substantial change in serum TSH levels was found in those children who did not lose weight. It should be highlighted that, while in our study the patients who experienced a failure of the intervention program were characterized by a significant body weight gain, the children included in the study by Reinehr et al. showed stable body weight at the end of follow-up. Several studies showed that isolated hyperthyreotropinemia due to obesity can revert after weight loss either achieved by the dietary intervention [8–11, 14–16] or bariatric surgery [12, 13, 17]. The specific design of the present study (all patients enrolled displayed normal TSH levels), allows to observe that a significant reduction in serum TSH occurs also in obese patients with normal TSH levels who experience weight loss. A potential limitation of the present study could be represented by the fact that adherence to the program was not evaluated through structured questionnaires. The here reported significant decrease in serum TSH, although of little magnitude, might not be free of relevant consequences, in that the TSH decrease was previously identified as a potential cause of the difficulties in maintaining weight loss. Previous studies [8, 18] demonstrated that the decrease in serum TSH during diet was positively correlated with weight regain after stopping the dietary regimen which appears in line with studies performed in adults and children reporting a decrease in energy expenditure in parallel with weight loss. Unfortunately, the design of the present study does not allow evaluating the issue of body weight regain.

A further finding of the present study is that higher basal TSH levels were observed in patients from the Success Group as compared to those of the Failure Group. This finding was strengthened by the logistic regression analysis, showing that differences in basal TSH were significantly and independently related to the outcome of the dietary program. These data are in line with what reported in a recent study [18] showing that baseline TSH levels were significantly higher in children who experienced a substantial weight loss as compared to those experiencing failure after a 1 year dietary intervention program. The here reported differences in baseline TSH levels between the two groups may be regarded as slight but, it should be considered that the presence of abnormal values of serum TSH constituted a mandatory exclusion criterion. The interplay between thyroid function and obesity is a widely investigated one. According to previous studies, the concept arose that elevated TSH levels, commonly observed in obese patients, would not be always indicative of subclinical hypothyroidism [4, 5]. Furthermore, some authors proposed that this increase in TSH levels should be regarded as an adaptation process to increase REE and, in turn, energy expenditure [1]. The clinical implications of these findings would require specifically designed studies, however, previous studies reported that TSH levels can normalize in obese patients following weight loss [14–17] and thyroxine administration in obese patients with moderately elevated TSH levels did not modify their body weight status [19].

Taken together the above concepts could explain the difference in basal TSH serum levels in obese patients according to success or failure of the dietary intervention program. It is interesting noting that a significant reduction in total cholesterol levels was observed in both groups of obese patients independently of the success/failure of the program, which could indicate that an attempt to modify eating habits was made by all the involved patients regardless of the final outcome. Indeed, data in the literature indicate that although there is a direct correlation between weight loss and reduction in total cholesterol levels [20], a low-fat diet can induce a reduction in total cholesterol even when bodyweight loss is limited [21].

The above concept could be potentially relevant in that it could suggest the hypothesis that success in a lifestyle intervention and/or in weight regain could not only be due to the compliance of obese patients, but also influenced by other factors, possibly including thyroid function. The results of the present study are not sufficient to confirm this intriguing hypothesis, and future, prospective studies will be needed to confirm it.

In conclusion, the results of the present study demonstrate that significant differences in both baseline and longitudinal behavior of serum TSH characterize patients with a different response to a dietary intervention program. Future longitudinal prospective studies will be required to establish firm conclusion as to the effect of TSH changes on weight regain as well as on the physiopathologic mechanism involved in this association.

### What is already known on this subject?

Hyperthyreotropinemia is a frequent finding in obese subjects. Substantial variations in thyroid function parameters are observed after body weight loss due to dietary intervention or bariatric surgery.

### What your study adds?

Differences in baseline and longitudinal behavior of serum TSH levels characterize obese patients with success or failure of a dietary intervention program.
